# Supplementary material for: The Role of GeneXpert® for Tuberculosis Diagnostics in Brazil: An Examination from a Historical and Epidemiological Perspective
Source: Trop Med Infect Dis. 2023 Oct 26;8(11):483. doi: 10.3390/tropicalmed8110483 (PMC10674801; doi:10.3390/tropicalmed8110483)
Supplement: Supplementary file 1 [file tropicalmed-08-00483-s001.zip › Table S1.pdf]

Supplementary Table S1. Trend analysis of the historical of *Mycobacterium tuberculosis (*MTB) and rifampicin (RIF)-resistant frequency series from 2014 to2020 by Brazilian region.

**Region**	**MTB Frequency**					**RIF-Resistance MTB Frequency**				
	**A**	**INTERPR**	**X²**	**GL**	**P-value**	**A**	**INTERPR**	**X²**	**GL**	**P-value**
**N**	3367,69	Increasing	215,25	1	<0.0001	-149,08	Decreasing	8,9065	1	0,0028
**NE**	-1198,89	Decreasing	12,58	1	0,0004	-21,38	Not applied	0,0804	1	0,7767
**SE**	3313,76	Increasing	37,75	1	<0.0001	-433,00	Decreasing	14,8746	1	0,0001
**S**	1875,85	Increasing	70,22	1	<0.0001	-70,78	Not applied	2,2855	1	0,1306
**CW**	554,91	Increasing	13,93	1	0,0002	436,22	Increasing	210,3732	1	<0.0001
**BR**	11167,98	Increasing	231,61	1	<0.0001	-225,86	Not applied	2,1219	1	0,1452

Information source: Elaborate by the authors, based on data from SINAN/CGDR/MS/IBGE, 2014-2020.
