# Supplementary figures and images for: The Role of GeneXpert® for Tuberculosis Diagnostics in Brazil: An Examination from a Historical and Epidemiological Perspective
Source: Trop Med Infect Dis. 2023 Oct 26;8(11):483. doi: 10.3390/tropicalmed8110483 (PMC10674801; doi:10.3390/tropicalmed8110483)

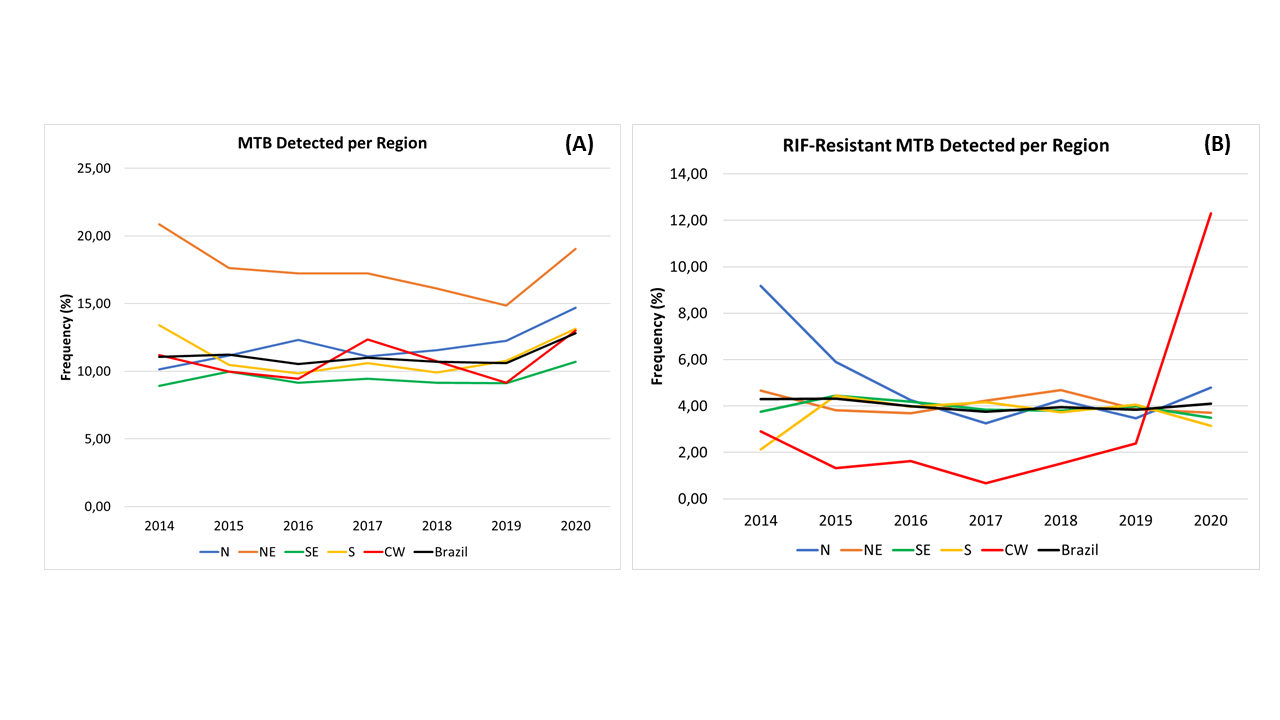

Supplement: Supplementary file 1 [file tropicalmed-08-00483-s001.zip › Figure S1.tif]
